# Molecular mechanism of K65 acetylation-induced attenuation of Ubc9 and the NDSM interaction

**DOI:** 10.1038/s41598-017-17465-0

**Published:** 2017-12-12

**Authors:** Mandar T. Naik, Mooseok Kang, Chun-Chen Ho, Pei-Hsin Liao, Yung-Lin Hsieh, Nandita M. Naik, Szu-Huan Wang, Iksoo Chang, Hsiu-Ming Shih, Tai-Huang Huang

**Affiliations:** 1 Institute of Biomedical Sciences, Academia Sinica, Taipei, 11529 Taiwan; 2 Department of Molecular Pharmacology, Physiology and Biotechnology, Brown University, Providence, Rhode Island 02903 USA; 3 Department of Physics, Pusan National University, Busan, 46241 Korea; 4 Center for Proteome Biophysics, Department of Brain and Cognitive Sciences, DGIST, Daegu, 42988 Korea; 5 Institute of Molecular and Genomic Medicine, National Health Research Institutes, Miaoli County, 35053 Taiwan

## Abstract

The negatively charged amino acid-dependent sumoylation motif (NDSM) carries an additional stretch of acidic residues downstream of the consensus Ψ-K-x-E/D sumoylation motif. We have previously shown that acetylation of the SUMO E2 conjugase enzyme, Ubc9, at K65 downregulates its binding to the NDSM and renders a selective decrease in sumoylation of substrates with the NDSM motif. Here, we provide detailed structural, thermodynamic, and kinetics results of the interactions between Ubc9 and its K65 acetylated variant (Ac-Ubc9_K65_) with three NDSMs derived from Elk1, CBP, and Calpain2 to rationalize the mechanism beneath this reduced binding. Our nuclear magnetic resonance (NMR) data rule out a direct interaction between the NDSM and the K65 residue of Ubc9. Similarly, we found that NDSM binding was entropy-driven and unlikely to be affected by the negative charge by K65 acetylation. Moreover our NMR, mutagenesis and molecular dynamics simulation studies defined the sequence of the NDSM as Ψ-K-x-E/D-x_1_-x_2_-(x_3_/E/D)-(x_4_/E/D)-x_n_ and determined that K74 and K76 were critical Ubc9 residues interacting with the negatively charged residues of the NDSM.

## Introduction

Post-translational modifications play a pivotal role in cell function due to their ability to rapidly and often reversibly change the behavior of the modified protein. Ubiquitin and ubiquitin-like modifications are unusual in that the modifiers themselves are small proteins^1^. Among these, modification by the small ubiquitin-like modifier (SUMO) has great significance in the regulation of various cellular processes, such as nuclear transport, transcription, chromosome segregation, and DNA repair^2^. At least four paralogues of SUMO exist in vertebrates; among these, SUMO-1 shares roughly 50% identity with SUMO-2/3. Sumoylation of substrate involves enzyme-mediated isopeptide bond formation between the C-terminus of mature SUMO and the side-chain of a target lysine residue, often, but not always defined by the sumoylation motif (SM), Ψ-K-x-E/D, where Ψ is a large hydrophobic residue^3,4^. The native SM can exist in different configurations, such as an inverted motif, hydrophobic cluster motif, and a negatively charged amino acid-dependent sumoylation motif (NDSM)^5,6^. Sumoylation appears to be a highly dynamic yet tightly controlled process whereby a steady-state level of the sumoylated protein is maintained by a cascade of three conjugating (E1, E2, and E3) and multiple de-conjugating enzymes^2^. Unlike the ubiquitin pathway, the SUMO pathway has only one known activating enzyme (E1), the dimeric SAE1/SAE2 complex, and a single conjugating enzyme (E2), Ubc9. The E3 ligases are not always essential for sumoylation. Ubc9 is a globular protein^7,8^, which facilitates transfer of SUMO from the E1 to the substrate with or without the help of E3. Ubc9 is implied to be involved in an adverse set of interactions with E1^9–11^, E3^12–16^, and the substrate^7,12–14,17^. Ubc9 recognizes the SM and is partly responsible for the stringency exhibited by the SUMO pathway. Ubc9 is known to have three different interactions with the SUMO moiety. Besides formation of a transient SUMO thioester intermediate on its active site C93 residue^18^, it can non-covalently bind SUMO using a separate interface on the opposite face of its active site^19,20^ and is itself a substrate for SUMO modification at K14^21^. Recently, Ubc9 has been shown to form homodimers^22^.

A serine residue downstream of the SM leads to tighter binding with Ubc9 after phosphorylation of the phosphorylation-dependent sumoylation motif (PDSM), Ψ-K-x-E/D-x_1_-x_2_-S-P^17^. Thus, PDSM is a convertible variant of the NDSM, in which the phosphorylated residue is substituted permanently by one or more acidic residues. The high resolution structure of the PDSM bound to Ubc9 is not available, but an extensive analysis of Ubc9 mutants shows that the basic patch formed by K65, K74, and K76 is required for discriminating phosphorylation of the PDSM^17^. Among the three residues, K65 and K74 are more important for recognition of the PDSM. Alanine substitution on all three residues leads to loss in the ability of Ubc9 to differentiate the phosphorylated substrate, but the K65 mutation does not compromise the conjugation activity of the enzyme, unlike the K74 and K76 mutations. Moreover, the K65 residue contributes less to NDSM recognition compared to its role in discriminating phosphorylated PDSM from the non-phosphorylated version^17^. The NDSM is defined as Ψ-K-x-E/D-(x/E/D)_n_, with a very wide variation in length and composition of the acidic stretch among various NDSM sequences^6^. To the best of our knowledge, only a limited biophysical characterization has been performed on NDSM substrates with debate on the roles of the K59 and R61 residues^6,17^, and no structural information is available. We have previously shown that acetylation of Ubc9 residue K65 serves as a dynamic switch for NDSM substrate sumoylation. We found that K65 acetylation downregulates Ubc9 binding to substrates with the NDSM but not to substrates carrying the consensus SM or SIM. Thus, Ubc9 K65 acetylation may exert selective control on sumoylation of the NDSM class of substrates, as demonstrated by us in the hypoxic response through the SIRT1/Ubc9 regulatory axis^23^. In the current study, we present our new findings describing the biophysical and structural characterization of the interaction between Ubc9 and the NDSM. We also explain the molecular mechanism beneath reduced binding between Ac-Ubc9_K65_ and the NDSM.

## Results

### NMR characterization of the interaction between NDSM, Ubc9, and Ac-Ubc9_K65_

**Figure 1:**
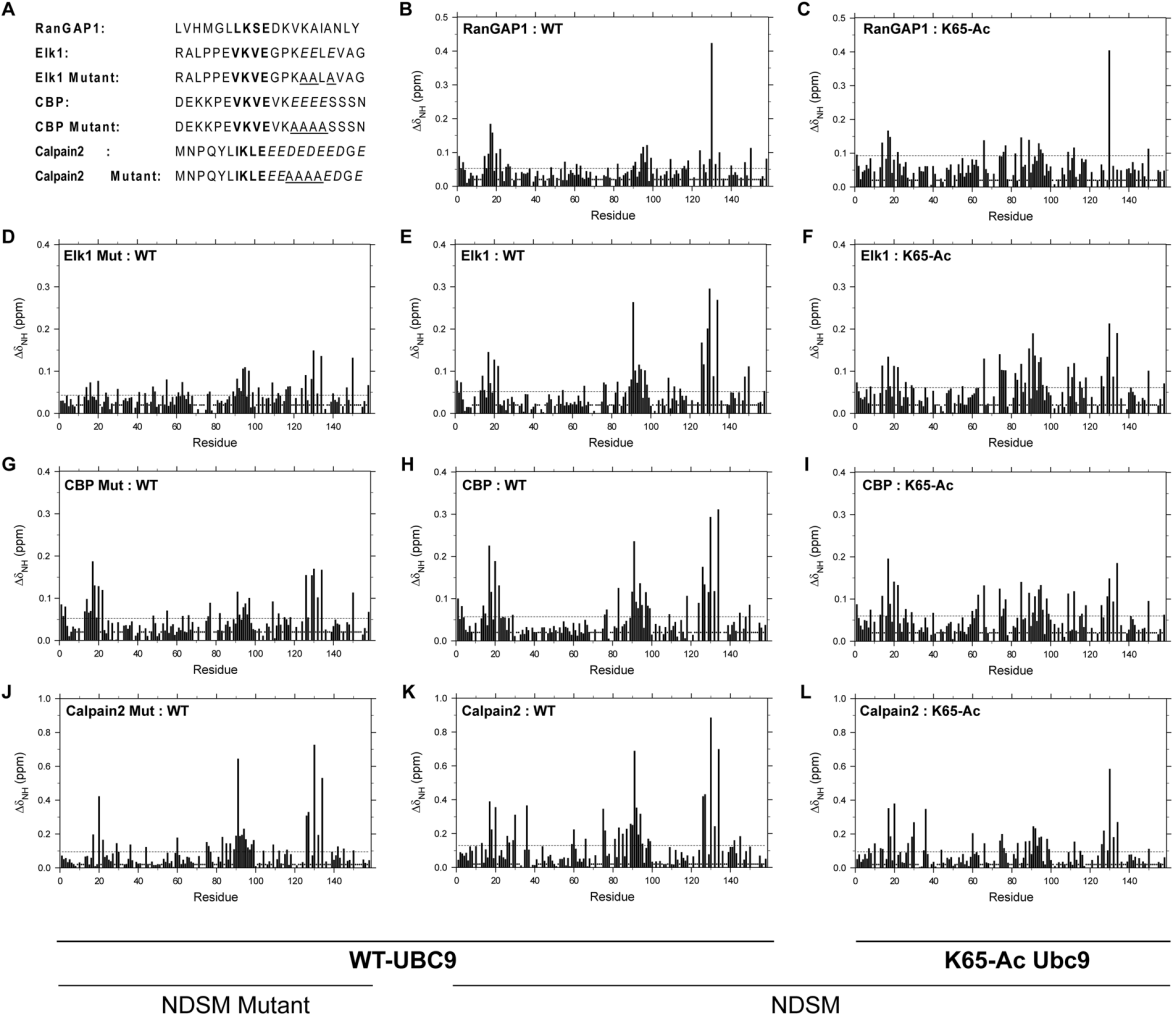
Nuclear magnetic resonance interaction study between Ubc9 and various peptides. Sequences of various peptides used in this study are shown in panel A. Average perturbation in the backbone amide chemical shifts of the Ubc9 residues upon binding of the RanGAP1 (**B** and **C**), Elk1 (**E** and **F**), CBP (**H** and **I**), and Calpain2 (**K** and **L**) derived peptides to the wild type and K65 acetylated proteins, respectively. Also shown are perturbations upon binding of the Elk1 (**D**), CBP (**G**), and Capain2 (**J**) mutant peptides. The data were acquired at 1:20 molar excess of the peptide. Dotted line shows average perturbation and asterisk marks missing data from a particular experiment.

We have reported previously^23^ that acetylation of Ubc9 K65 attenuates the NDSM substrate interaction and its subsequent sumoylation. Here, we used various biophysical techniques to elucidate the structural basis for this effect. It has been previously shown that Ubc9 recognizes the ΨKxE SM sequence. The chemical shift perturbation induced in Ubc9 by binding of the peptides derived from p53 and c-Jun is subtle but specific to the continuous binding interface on Ubc9^8^. This moderate perturbation can be attributed to the four short ΨKxE stretch residues of the SM that result in limited contact with Ubc9 due to the buried surface area of ~750 Å^2^. High resolution atomic details of this interaction can be seen in the crystal structure of Ubc9: RanGAP1 protein complexes^7,14^. We decided to first reproduce these observations in solution using a short peptide depicting the RanGAP1 SM (Fig. 1A). NMR HSQC spectra were acquired on free- and peptide bound-Ubc9 to follow the residue-specific binding change. Pronounced changes were observed in three regions of Ubc9, such as the hydrophobic pocket, the active site, and around the N-terminal sumoylation site (Fig. 1B). Residue Q130, which was perturbed the most, lies at the hydrophobic pocket where the hydrophobic residue from SM, Ψ, binds, whereas residues around C93 formed the Ubc9 catalytic active site. The specific chemical shift perturbations of the residues in these two regions were consistent with the interaction surface seen in the crystal structure^14^ (Fig. 1). The perturbation seen around Ubc9 self-sumoylation site K14 from the first N-terminal helix is likely an indirect manifestation of interference with Ubc9 homodimerization due to substrate binding^22^. We also observed this indirect perturbation in all peptides used in our study and do not foresee this to be a direct interaction interface with the sumoylation substrate (Fig. 1).

Interestingly, a very similar perturbation pattern was induced by binding of the RanGAP1 peptide to the acetylated Ubc9 protein; however, distinctly strong changes were seen around the acetyllysine location, particularly in the next residue D66, then residues from the neighboring β-strand, including K74, C75, K76, and F77; and finally residues N85 and S89 (Fig. 1C). We then studied three NDSM peptides derived from the Elk1, CBP, and Calpain2 proteins (Fig. 1A). All of the NDSM peptides induced a stronger perturbation in Ubc9 compared to that in the RanGAP1 consensus motif (Fig. 1E,H and K). The overall perturbation pattern observed in the Ψ binding pocket as well as near the Ubc9 active site suggests that the NDSM binds Ubc9 similar to the RanGAP1 consensus motif, but, more importantly, the NDSM induced a stronger perturbation in the aforementioned residues D66, C75, K76, and F77. Here, we could not obtain information for the important K74 residue due to spectral overlap. Interestingly, only a feeble perturbation was seen in the backbone amide of the K65 residue.

We then studied the effect of acidic residues that differentiate the NDSM from the consensus sumoylation motif. We synthesized alanine mutant peptides by replacing every acidic residue appearing in four positions after Ψ-K-x-E-x_1_-x_2_- (Fig. 1A). Such NDSM mutant peptides lost the ability to perturb the additional Ubc9 residues mentioned above (Fig. 1D,G and J). The perturbation induced in the Ubc9 active site by the acidic residue mutant peptides derived from Elk1 and CBP then closely resembled the RanGAP1 consensus motif. A significantly less prominent change was observed in the acidic residue mutant peptide derived from CALPAIN2, as not all acidic residues from the original sequence were substituted with alanine. Overall, the NDSM acidic residues can be postulated to interact with the shallow positively charged binding surface around the K74 and K76 residues. We next studied binding of various NDSM peptides with Ac-Ubc9_K65_ (Fig. 1F,I and L). Although NDSM bound the acetylated protein, the profile clearly showed less perturbation in the specific Ψ binding pocket residues and those in the Ubc9 active site. Thus, these results suggest that the NDSM binds weakly to Ac-Ubc9_K65_.

### Thermodynamics of NDSM binding to Ubc9 and Ac-Ubc9_K65_

**Figure 2:**
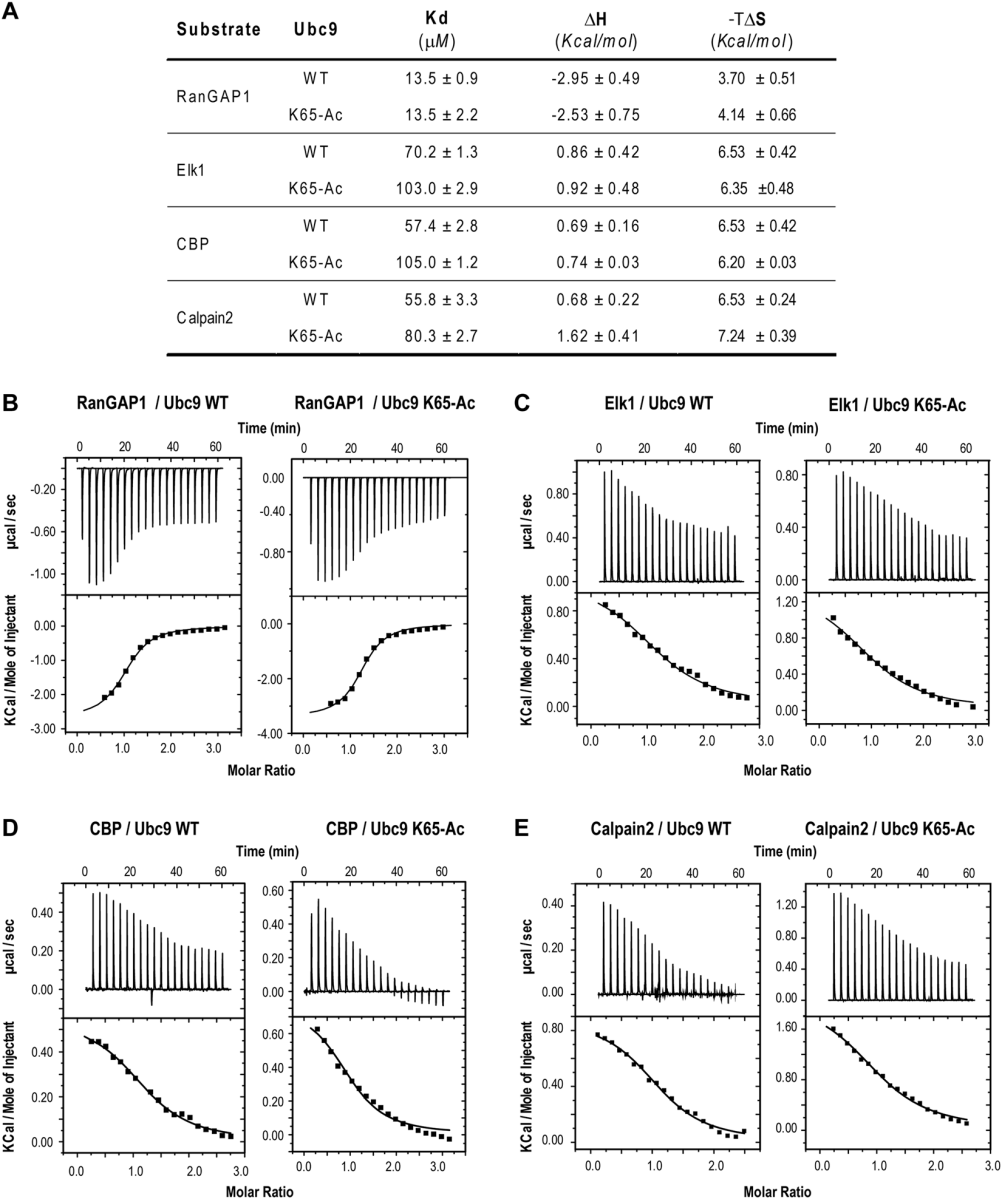
Effect of K65 acetylation on Ubc9 affinity for substrates. (**A**) Summary of the thermodynamic parameters obtained from triplicate isothermal titration calorimetry (ITC) measurements. Representative ITC thermograms for Ubc9 binding to the RanGAP1 (**B**), Elk1 (**C**), CBP (**D**), and Calpain2 (**E**) peptides. The heat absorbed (or released) post-injection, after correcting for the heat of dilution, is shown in lower panels.

ITC binding experiments were used to quantify the effect of Ubc9 acetylation on NDSM substrate binding (Fig. 2A). Our data show that contrary to the exothermic binding equilibrium obtained for the consensus RanGap1 motif (Fig. 2B), various NDSM peptides bound endothermically to the WT Ubc9 protein by reacting with a significantly increased entropy change. The dissociation constants for Elk1 and CBP were 70 and 57 μM, respectively (Fig. 2C and D), which were roughly similar to the dissociation constant of 56 μM obtained for the more acidic Calpain2 (Fig. 2E). Contrary to our expectations, RanGAP1 bound more strongly than the NDSMs with a dissociation constant of 13 μM. This observation suggests that the amino acid composition of the sumoylation substrate, particularly the type and likely the conformation of residue Ψ, is of paramount significance to determine its binding affinity for Ubc9. Similarly, not every acidic residue from the NDSM contributed equally to Ubc9 binding and even positions ×1 and ×2 could be potentially deciding factors. We exploited the long acidic stretch of Calpain2 in additional studies described below to explain this phenomenon. Additionally, the dissociation constant of the Ac-Ubc9_K65_ interaction with the Elk1, CBP, and Calpain2 NDSM peptides (Fig. 2C–E) increased significantly to 103, 105, and 80 μM, respectively. These data directly demonstrate that K65 acetylation reduced the binding affinity between the Ubc9 and NDSM peptides by 1.5–1.8 fold. In contrast to the NDSM peptides, the RanGAP1 peptide bound exothermically and yielded a comparable dissociation constant (13 μM) with those of the WT and Ac-Ubc9_K65_ proteins (Fig. 2B). Thus, acetylation of K65 did not affect binding of the consensus SM and modulated binding of only longer variants, such as the NDSM. The observed switching from exothermic versus endothermic binding of the NDSM versus SM containing peptides is surprising. We speculate that the long negatively charged tail of NDSM may interact with Ubc9 in such a way that it free up more Ubc9-bound water molecules, resulting in an overall entropy increase. More work need to be done to fully understand its implication in binding mechanism.

### Ubc9 K65 acetylation affects K_m_, but not K_cat_ for the NDSM

**Figure 3:**
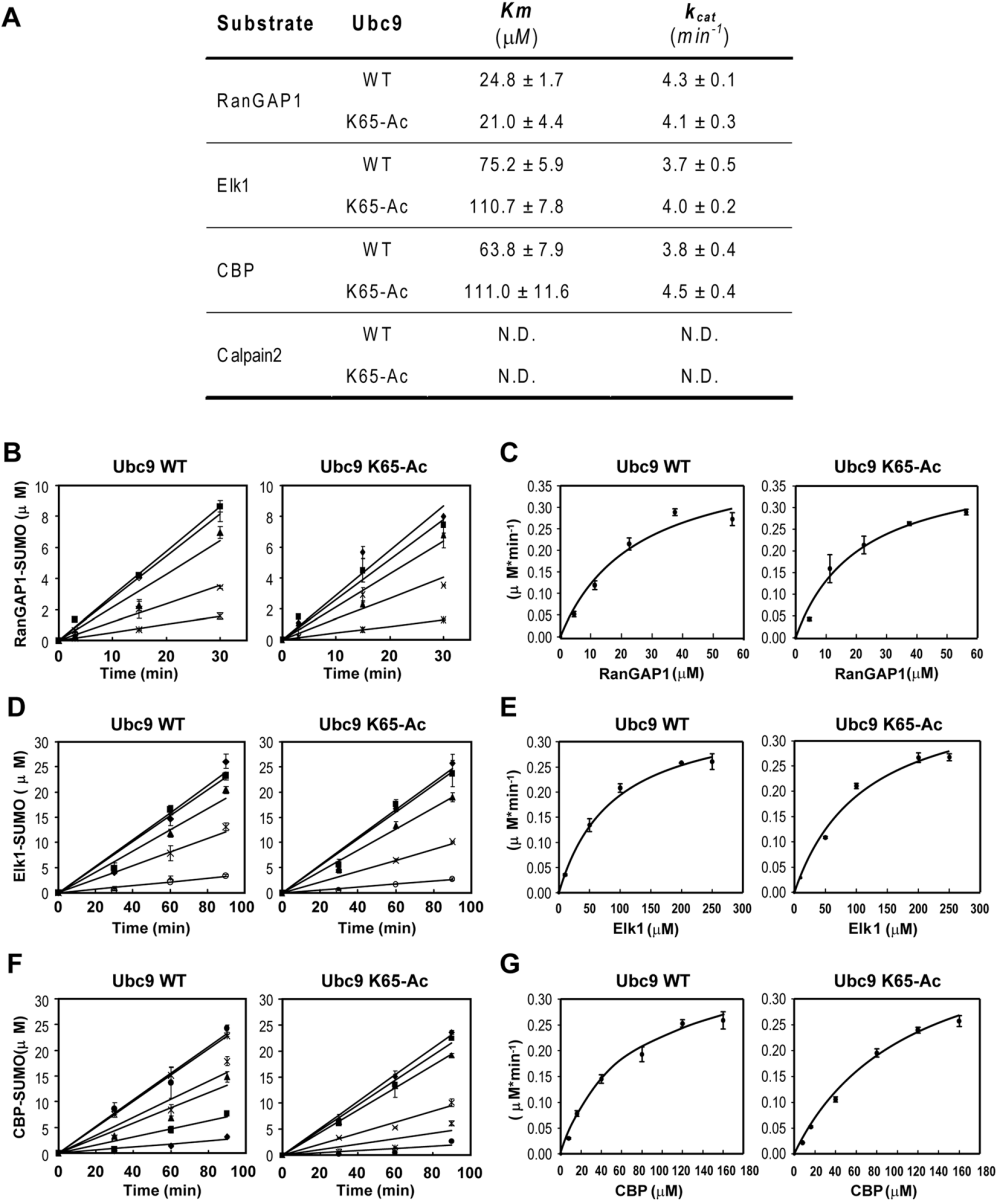
Effect of K65 acetylation on Ubc9 catalysis. (**A**) Summary of Michaelis–Menten kinetics applied to SUMO conjugation rates for GST-RanGAP1_481-587_ (**B** and **C**), GST-Elk1_200-261_ (**D** and **E**) and GST-CBP_1000-1080_ (**F** and **G**). Experiments performed in triplicate.

Although the decreased Ubc9 binding to the NDSM substrates on K65 acetylation could explain the reduced levels of NDSM substrate sumoylation^23^, we could not entirely exclude the possibility that K65 acetylation could also affect Ubc9 catalytic activity. To test this possibility, an enzyme kinetics study was performed using multiple-turnover *in vitro* sumoylation assays between WT or Ac-Ubc9_K65_ and GST fusion of SM derived from RanGAP1 (Fig. 3A and B), Elk1 (Fig. 3C and D), and CBP (Fig. 3E and F). Notably, WT and Ac-Ubc9_K65_ gave similar *k*_cat_ values for RanGAP1, Elk1, and CBP, but higher *k*_m_ values for Elk1 and CBP than that of RanGAP1 (Fig. 3A). In line with the ITC results, K65 acetylation increased the *k*_m_ values by 1.5–1.7 fold of the NDSM peptides, but not the RanGAP1 SM. These results suggest that K65 acetylation mainly affects Ubc9 binding affinity toward the NDSM substrates, rather than altering Ubc9 catalytic activity.

### K65 acetylation remodels the NDSM recognition site without being involved in direct binding

**Figure 4:**
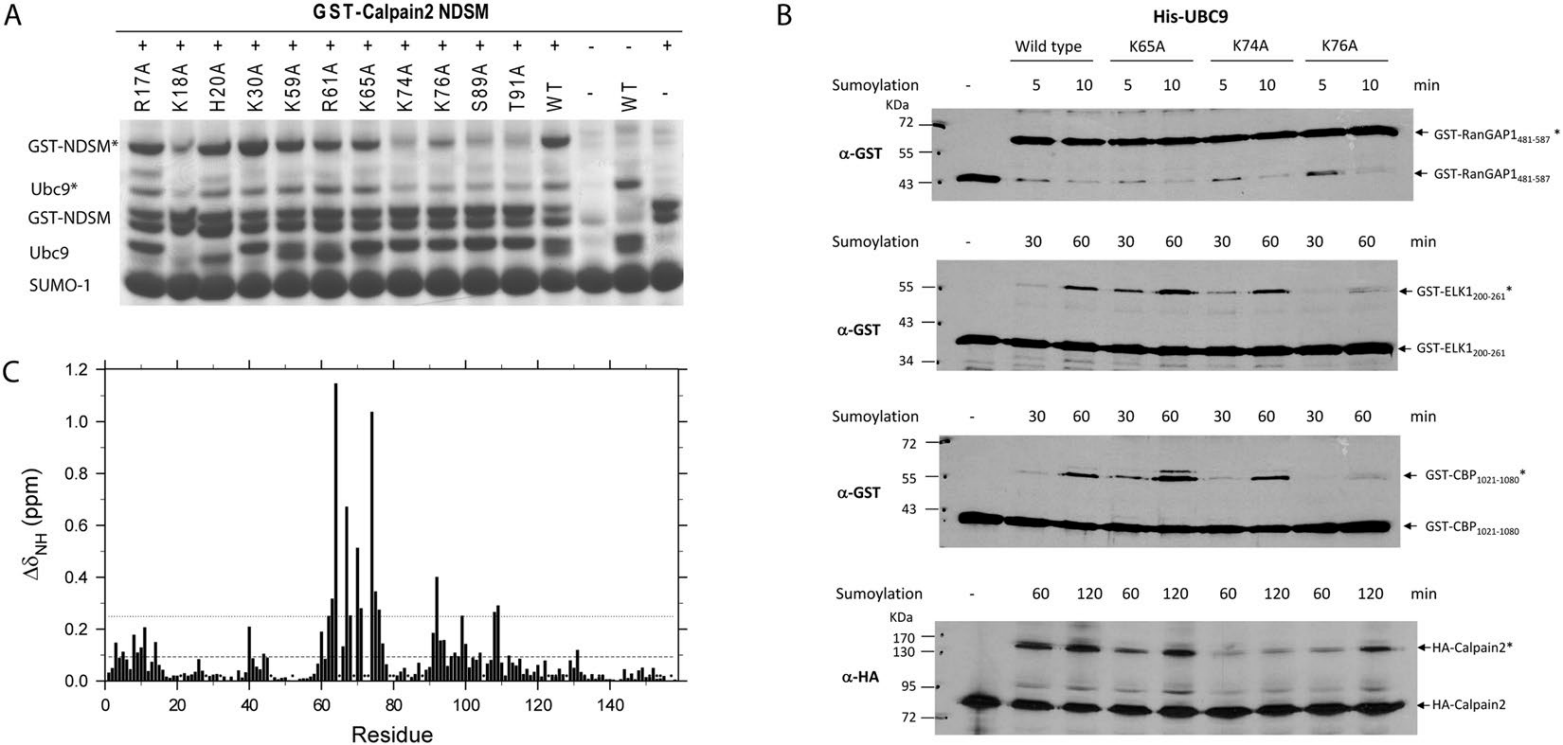
Effect K65 acetylation on Ubc9 structure. Sodium dodecyl sulfate-polyacrylamide gel electrophoresis analysis of sumoylation assays performed using (**A**) GST-Capain2_385-406_ and various single alanine mutants of Ubc9. “*” Denotes sumoylated species. (**B**) Western analyses of *in vitro* sumoylation reactions of GST-RanGAP1, Elk-1, and CBP domain recombinant proteins, and *in vitro* synthesized HA-tagged Calapin2 protein with indicated Ubc9 WT and mutant proteins. (**C**) Residue specific average chemical shift perturbation in Ubc9 backbone amides upon acetylation of K65.

We then performed Ubc9 alanine point mutations to delineate important residues involved in binding with the NDSM. Eleven Ubc9 mutants were prepared throughout the SM binding interface identified in various crystal structures^17^. Sumoylation of the GST-Calpain2 NDSM was chosen for this study first as it has the most extensive stretch of acidic residues among all NDSMs known to date (Fig. 4A)^6^. As expected, residues around the S89 and T91 active sites are critical for recognition and sumoylation of the NDSM but residues from the N-terminal region, R17, K18, H20, and K30 are not important. Among the lysine residues, K74 and K76 are critical but the remaining lysine and arginine residues, including K59 and R61, and the acetylation modification site, K65 are not important. We then studied the effect of Ubc9 K65A, K74A and K76A mutations on their interactions with RanGAP, Elk1 and CBP. Similarly, K76 but not K65 is important for sumoylation of Elk-1 and CBP (Fig. 4B). However, Ubc9 K74 mutation has little effect on the sumoylation of Elk1 and CBP. Importantly, none of the mutations has any effect on RanGAP sumoylation. This study further validates our NMR observations that the K65 acetylation site is not directly involved in the interaction with the NDSM. Notably, K65 acetylation induced a significant chemical shift perturbation in the Ubc9 backbone (Fig. 4C). Among the strongest perturbed residues by adding the acetyl moiety were F64 and K74. As there is no clear definition available for the NDSM, it was denoted as Ψ-K-x-E/D-(x/E/D)_n_. Our studies suggest that not every position of the acidic stretch contributed equally to Ubc9 binding, so we numbered each amino acid after the E, as Ψ-K-x-E/D-x_1_-x_2_-x_3_-x_4_-x_5_-x_6_-x_7_-x_8_-x_9_-x_10_ for simplicity.

### Molecular dynamic simulations of the NDSM binding to Ubc9 and Ac-Ubc9_K65_

**Figure 5:**
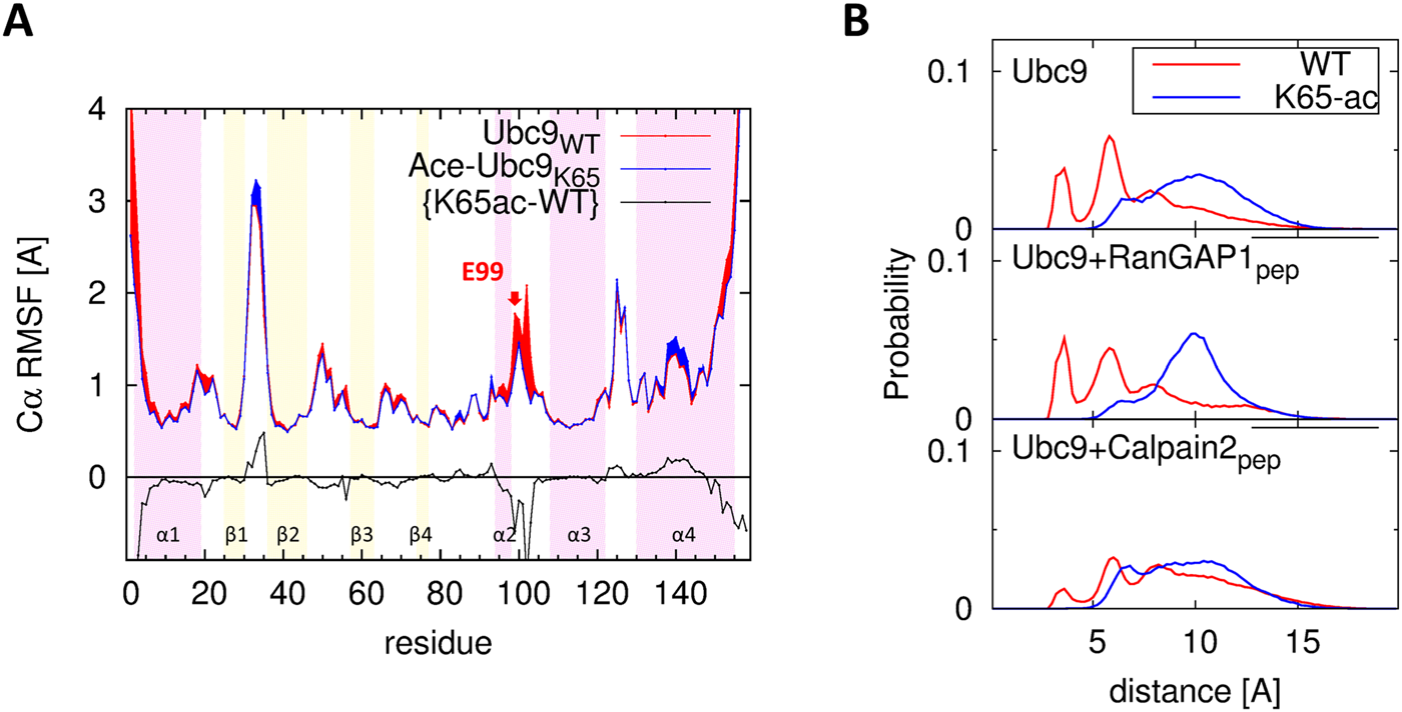
Structural changes of Ubc9 upon K65 acetylation. (**A**) Cα RMSF of Ubc9_WT_ (red line) and Ac-Ubc9_K65_ (blue line). The difference in RMSF between WT and K65-ac is denoted by a black line. Background color denotes secondary structure (purple: α-helix, yellow: β-strand). (**B**) The distance (d_K65_-_E99_) distribution between K65 and E99 in Ubc9_WT_ (red) and Ac-Ubc9_K65_ (blue). Ubc9 (top), Ubc9-RanGAP1_peptide_ complex (middle) and Ubc9-Calpain2_peptide_ complex (bottom).

We performed MD simulations with the Ubc9_WT_ and Ac-Ubc9_K65_ structures to gain better insight into their binding to the NDSM. We first analyzed the structural difference between free Ubc9_WT_ and free Ac-Ubc9_K65_. Although no significant change in backbone structure was detected, the side-chain interaction between K65 and E99 disappeared upon K65 acetylation, which was reflected by the major difference in RMSF of α2-α3 loop (98–103) including E99 (Fig. 5A). We took the distance (d_K65_-_E99_) distribution of the hydrogen bond interactions between the Nζ atom of K65 and the Cδ atom of E99 as a reference measure to investigate the effect of K65 acetylation as well as that before and after binding with RanGAP1 and Calpain2. The d_K65_-_E99_ in a free Ubc9_WT_ (top panel of Fig. 5B) distinctively peaked around the typical values of hydrogen bond interactions, whereas d_K65_-_E99_ in a free Ac-Ubc9_K65_ was shifted toward larger values and was much more broadly distributed. Thus, K65 in a free Ubc9_WT_ (Ac-Ubc9_K65_) was mostly (not) in the proximity of E99, and acetylation of K65 prevented it from attracting E99. The d_K65_-_E99_ distributions for the Ubc9-RanGAP1 complex (middle panel of Fig. 5B) were similar to those of free Ubc9_WT_ and free Ac-Ubc9_K65_, suggesting that K65 in the Ubc9-RanGAP1 complex and its acetylation do not interfere with the binding of RanGAP1. In contrast, the d_K65_-_E99_ distribution of the Ubc9_WT_-Calpain2 complex (red curve in bottom panel of Fig. 5B) peaked much less around the typical value of the hydrogen bond interaction and was broadly distributed together with the blue curve for that of the Ac-Ubc9_K65_-Calpain2 complex. This finding suggests Ubc9-Calpain2 interaction remodel the Ubc9 structure such that that K65 is no longer hydrogen bound to E99 in either the wild-type or K65 mutant form.

The binding affinities between Q130, D127, K74, S89, and T91 of Ubc9 and the SM ΨKxE of RanGAP1, Elk1, and Calpain2 were estimated using the structural ensembles obtained by our MD simulations to clarify the structural nature of the different degrees of interaction caused by K65 acetylation across the binding interface between Ubc9 and the binding substrates. The hydrophobic interactions across the binding interface were probed by the distance between Ψ and Q130. A similar distance distribution was observed in the binding structures of RanGAP1 and Calpain2 with both Ubc9_WT_ and Ace-Ubc9_K65_ (Table 1). We analyzed every MD trajectory of Elk1, as the Ac-Ubc9_K65_-Elk1 complex showed more than a 10 Å distance for Ψ-Q130, and we observed that Elk1 did not have a stable binding structure with Ac-Ubc9_K65_. The hydrogen-bond occupancy between K and D127 for RanGAP1 and Calpain2 bound with both Ubc9_WT_ and Ace-Ubc9_K65_ also showed similar results, although K65 acetylation promoted a slight increase during this occupation. Therefore, the hydrophobic interaction of Ψ-Q130 and the hydrogen-bond interaction of K-D127 are not the determining factors for differentiating the downregulating effect of K65 acetylation. We further investigated the hydrogen-bond interactions between E and K74 and S89 and T91. The hydrogen-bond occupancies for these pairs in the Ac-Ubc9_K65_-RanGAP1 (Calpain2) complex were the same or higher (lower) than those in Ubc9_WT_–RanGAP1 (Calpain2) complex. Of all the differences, the hydrogen-bond occupancy of E-K74 in the Ac-Ubc9_K65_-Calpain2 complex was more than two-fold weaker than that in the Ubc9_WT_-Calpain2 complex. This observation suggests that K74 is a determining residue for exhibiting the downregulating effect of K65 acetylation in Ubc9 when binding with Calpain2, but not with RanGAP1 (Table 1).

**Table 1:** Binding affinities between Q130, D127, K74, S89, T91 of Ubc9 and the ΨKxE sumoylation motif of RanGAP1, Elk1, and Calpain2 substrates.

Substrate	Ubc9	Ψ388-Q130 Distance (A)	Hydrogen bond occupancy (%)			
			K389-D127	E392-K74	E392-S89	E392-T91
RanGAP1	WT	8.0 ± 0.7	70.6	46.7	83.71	75.8
	K65-ac	7.7 ± 0.4	75.5	49.0	93.5	97.1
Elk1	WT	8.7 ± 0.6	71.7	74.9	89.8	94.52
	K65-ac	N.D	N.D	N.D	N.D	N.D
Calpain2	WT	8.0 ± 0.5	76.8	79.7	92.3	97.7
	K65-ac	8.1 ± 0.8	84.2	32.7	79.7	80.6

**Figure 6:**
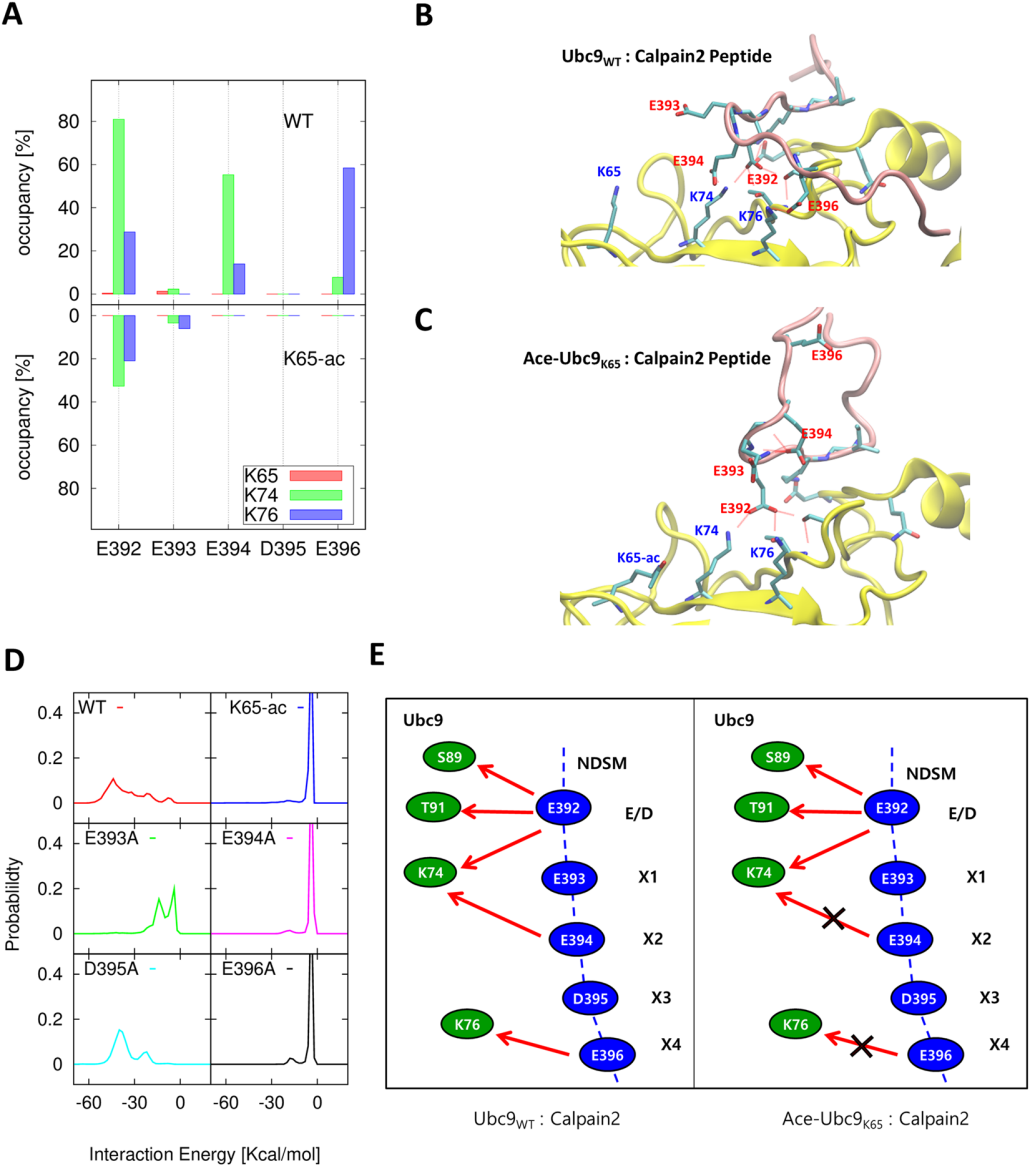
Direct interaction between Ubc9 and negative residues of the NDSM. (**A**) Hydrogen bond occupancy between K65, K74, and K76 in Ubc9 and E, ×1– ×4 residues in the Calpain2 peptide. (**B**) Binding interface between Ubc9_WT_ and the Calpain2 peptide. (**C**) Binding interface between Ac-Ubc9_K65_ and the Calpain2 peptide. (**D**) The distribution of interaction energy between K74-C75-K76 in Ubc9 and ×1– ×4 residues in the Calpain2 peptide. (**E**) Schematic for the change that occurs in the network of hydrogen-bond interaction (red arrows) between Ubc9 (green circle) and Calpain2 (blue circle) upon K65 acetylation.

We calculated the hydrogen-bond occupancy between K65, K74, and K76 of Ubc9 and ×1, ×2, ×3, and ×4 of Calpain2 to further investigate the role of negatively charged residues in the NDSM (Fig. 6A). As expected, neither K65 formed a hydrogen-bond interaction with the NDSM, supporting the view for the indirect involvement of K65 in Ubc9-Calpain2 binding. The hydrogen-bond interactions K74-E392, K74-(×2)E394, and K76-(×4)E396 in the Ubc9_WT_-Calpain2 complex were significant. In contrast, the K74-E392 interactions in theAc-Ubc9_K65_-Calpain2 complex became weaker by more than two-fold, and the K74-(×2)E394 and K76-(×4)E396 interactions disappeared. Therefore, we concluded that K74-E392, K74-(×2)E394, and K76-(×4)E396 are the determining pairs of residues that manifest the effect of K65 acetylation (Fig. 6B and C). We compared these MD simulation results from the HADDOCK and Calpain2-domain based models and both demonstrated consistent results. These results were also confirmed by the distribution of interaction energy between K74-C75-K76 of Ubc9 and (×1)E393–(×4)E396 of Calpain2 for the Ubc9_WT_-Calpain2 complex, as well as the Ac-Ubc9_K65_-Calpain2 complex and the ×1– ×4 mutants in the NDSM (Fig. 6D). The distribution curves illustrate the presence of dominant conformations in the WT, near −50 Kcal/mol with the hydrogen bond interactions of both K74-(×2)E394 and K76-(×4)E396. However, none of these hydrogen bond interactions were present in the case of K65-ac. The ×2 A(E394 A) and ×4A(E396A) mutants lost all of their interactions, like K65-ac. E394 interacted only with K74 in the ×1 A(E393 A) mutant, but the interaction was weak. The ×3A(D395A) mutant did not change binding stability. We also found that when ×2 and ×4 were mutated to alanine, ×1 and ×3 could not replace the role. Figure 6E presents a schematic for the change that occurred in the hydrogen-bond interaction network between Ubc9 and Calpain2 upon K65 acetylation.

We performed additional MD simulations for the Ubc9 complex with the Elk1 and CBP peptides. We made the initial peptide structure from the domain structure of Calpain2. However, we could not create a structure for the Elk1 and CBP cases, because no three dimensional Elk1 and CBP structural information was available. Thus, we constructed the initial peptide structure of Elk1 and CBP starting from its stretched peptides. We applied NMR restraint to make a docked complex with Ubc9. Then, we performed additional MD simulations under the same conditions for Calpain2 and clustered the MD trajectories. The hydrogen-bond occupancy between Ubc9 and the NDSM was estimated (see Supplementary Table 1). The results showed that, as expected, for Elk1 and CBP ×2 does not form hydrogen bond with any of the Ubc9 residues since it is not a negatively charged residue. In CBP, high hydrogen-bond occupancies for ×4-K74 and ×4-K76 pairs were found only in the complex with wild-type Ubc9 but not with the ac-Ubc9_K65_. Interestingly, in the Elk1-Ubc9 complex ×5 take up the role of ×4 found in both Calpain2 and CBP. We reason that the proline residue at the ×1 position may affect the conformation of Elk1 such that the ×5 residue is located in the position occupied by ×4 in CBP and Calpain2 peptides. Thus, the redundancy of the stretch of negatively charged residues in NDSM is important for its function. In summary, the MD simulation reaffirmed the role of the interaction between the stretch of NDSM negatively charged residues with Ubc9 K74 and K76 in modulating the sumoylation process and in exhibiting the Ubc9 K65 acetylation effect.

## Discussion

Our results indicate that the NDSM binds Ubc9 similar to the consensus SM but that there were additional contacts due to a more extensive interface. Interestingly, the NDSM binding was entropy-driven, which contradicted the common notion that charge-charge interactions between the NDSM and multiple lysine residues on the Ubc9 surface stabilize this binding. Reshuffling of water molecules on the interface alongside fine remodeling of the Ubc9 binding surface may be important for the outcome of NDSM binding. Moreover, the NMR data ruled out a direct interaction between the NDSM and the acetyllysine residue at position 65. Therefore, we performed extensive mutagenesis and NMR experiments to understand the effect of K65 acetylation on NDSM substrate binding to Ubc9.

Simple ^15^N- HSQC spectra were used to investigate the interaction between Ubc9 and the NDSM peptides from Elk1, CBP, and Calpain2. As a control, binding of the RanGAP1 peptide containing the ΨKxE motif to Ubc9 induced a prominent perturbation on the Q130 Ubc9 residue and a clustered moderate perturbation near the active C93 residue site (Fig. 1B). This perturbation was consistent with the interaction seen on the surface of the crystal structure^7,14^, suggesting that the hydrophobic residue Ψ binds near Ubc9 residues P128 to A131; the sumoylation target residue K contacts Y87, C93, and D127, whereas acidic residue E interacts with K74, S89, and T91. Of note, when the NDSM peptides bound to Ubc9, strong perturbations around Ubc9 residues Q130 and C93 were observed, such as T91 and residues Q126 to Y134 (Fig. 1E,H and K), which were distinct from the pattern shown by the RanGAP1 peptide. Interestingly, these perturbations decreased significantly when the NDSM acidic residue mutant peptides were used for Ubc9 binding (Fig. 1D,G and J), suggesting that the acidic residues within NDSM peptides are important to introduce tighter contact with Ubc9. More importantly, when the Ac-Ubc9_K65_ protein was assayed with the Elk1, CBP, or Calpain2 NDSM peptide, the perturbation at T91 and residues Q126–Y134 was reduced to a pattern similar to that of Ubc9 in the complex with the NDSM acidic residue mutant peptides (Fig. 1F,I, and L). In contrast, the perturbation pattern of Q130 and the residues around C93 induced by binding of the RanGAP1 peptide were preserved in the Ac-Ubc9_K65_ Ubc9 protein. These results suggest that K65 acetylation specifically alters NDSM peptide binding to Ubc9. It should be noted that none of the NDSM peptides studied by us induced a significant perturbation in the Ubc9 K65 residue, suggesting that K65 may not directly contact the stretched acidic residues within the NDSM peptides. We also observed that acetylation at K65 conferred significant chemical shift perturbations at Ubc9 residues F64, D67, S70, and K74 (Fig. 4B), which correlated with increased perturbation in residues near D66 and K74 in the binding study of the Elk1, CBP, and RanGAP1 peptides with the Ac-Ubc9_K65_ Ubc9 protein (Fig. 1F,I, and L). These data suggest that K65 acetylation likely contributes to a local conformational change in Ubc9, making it less favorable for NDSM peptide binding.

Our NMR data suggest that acetylation of K65 reduces the binding specificity of NDSM peptide towards Ubc9. The NDSM gain over the consensus motif due to stretch of the acidic residues is offset by acetylation of K65. Intriguingly, the actual acetylation residue, K65, did not appear to directly contact the NDSM peptide; thus, hinting at a possible indirect regulatory event. The chemical shift perturbation in the K65 acetylated protein with respect to the WT protein indicates significant remodeling of the basic surface for docking of the NDSM acidic patch. Of particular importance are the strong changes in and around K74. Our multiple-turnover kinetics measurements show that K65 acetylation mainly affected Ubc9 binding affinity toward the NDSM substrates, rather than altering Ubc9 catalytic activity. We then relied on extensive mutagenesis studies to identify the critical residues involved in Ubc9 binding and came to the conclusion that the NDSM can be defined as Ψ-K-x-E/D-x_1_-x_2_-E/D-x_4_-x_5_-x_6_-x_7_-x_8_-x_9_-x_10_. Our intensive MD simulations indicate that K65 acetylation caused weakening of the hydrogen-bond interactions between K74 and K76 with the negative NDSM residues located between ×2 and ×5 Overall, K65 acetylation resulted in attenuation of the binding affinity of Ubc9 to NDSM, such as Calpain2, Elk1, and CBP, but not with non-NDSM, such as RanGAP1. The interactions between Ubc9 K74 and K76 and the stretch of negatively charged residues between ×2 and ×5 are critical in NDSM sumoylation and in manifesting the effect of Ubc9 K65 acetylation.

## Methods

### Protein Preparation

Protocols for recombinant protein expression and purification were described previously^23^. Ac-Ubc9_K65_ was prepared by a strategy described elsewhere^24^. Briefly, E. Coli BL21 (DE3) transformed with pAcKRS-3 and pCDF PylT-1-Ubc9 with amber codon mutation at K65 positon, were grown in LB medium supplemented with kanamycin (50 μg/ml) and spectinomycin (50 μg/ml) at 37 °C till OD_600_∼0.7, then the culture was supplemented with 20 mM NAM and 10 mM acetyl-lysine followed in 30 minutes by the addition of 0.5 mM IPTG. Protein samples were purified by Ni-NTA chromatography with subsequent gel filtration on FPLC. Samples of wild-type (WT) Ubc9 and Ac-Ubc9_K65_ with a uniform ^15^N isotope enrichment were prepared using ^15^NH_4_Cl in the recipe for minimal M9 expression media. As preparing Ac-Ubc9_K65_ Ubc9 requires acetyllysine to be separately supplemented in the expression media, those samples lacked isotope labeling of the acetylated lysine residue. The synthetic peptides used in this study were purchased commercially.

### Biochemical Assays

All enzymes used for the biochemical assays were prepared by recombinant methods using plasmids described in earlier publications^23,25^ and stored at −80 °C prior to use. The GST-Ubc9 alanine mutants were prepared by site-directed mutagenesis. The *in vitro* sumoylation assay^26^ was performed in a 100 μl reaction mixture of 1 μM SUMO-1, 65 nM E1, 0.5 μM WT or mutant Ubc9, and 0.2 μM GST-Calpain2_385-406_ in buffer containing 20 mM HEPES (pH 7.3), 110 mM potassium acetate, 2 mM magnesium acetate, 1 mM EGTA, 1 mM DTT, and 0.05% Tween 20. All reactions were initiated simultaneously by adding 1 μl of 100 μM ATP followed by a 2 hour incubation at 30 °C. The reaction products were separated by sodium dodecyl sulfate-polyacrylamide gel electrophoresis (SDS-PAGE) and visualized with Coomassie Blue staining.

The kinetic analysis^27^ was performed in a 40 μl reaction mixture containing 10 μM SUMO-1, 150 nM E1, 100 nM WT or Ac-Ubc9_K65_, and known quantities of sumoylation substrate dissolved in a buffer containing 20 mM HEPES (pH 7.5), 100 mM NaCl, 5 mM MgCl_2_, and 2 mM ATP. The samples were subject to SDS-PAGE and visualized by SYPRO-RUBY staining using the UVP Gel-Doc-It system to quantify the sumoylated and non-sumoylated protein bands. Data from triplicate experiments were plotted as a graph of reaction velocity against concentration of the substrate to obtain the Michaelis–Menten constant (*k*_m_) and the turnover number (*k*_cat_).

### Isothermal titration calorimetry (ITC) measurements

The ITC experiments were repeated twice at 25 °C by a controlled 2 μl addition of peptide to 200 μl of WT or Ac-Ubc9_K65_ in pH 8.0 buffer containing 20 mM potassium phosphate and 100 mM NaCl using an iTC200 calorimeter (GE Healthcare, Parsippany, NJ, USA). The first data point of each experiment was excluded from analysis and the baseline-corrected data were analyzed using Origin 7.0 software assuming 1:1 binding stoichiometry.

### Nuclear magnetic resonance (NMR) spectroscopy

NMR data were acquired at 25 °C using software Topspin version 2.1 on a Bruker Avance 600 MHz spectrometer (Billerica, MA, USA) equipped with a cryogenic probe. Two dimensional-^15^N-heteronuclear single quantum coherence (2D-^15^N-HSQC) spectra were acquired on 0.2 mM Ubc9 or its 1:20 molar ratio complexes with various peptides dissolved in pH 7.0 buffer made up of 50 mM potassium phosphate, 100 mM NaCl, 5 mM β-mercaptoethanol and 7% D_2_O. Each spectrum was acquired as a matrix of 1024 ×128 complex points with spectral widths of 8,389 Hz and 1,235 Hz in the F2 and F1 dimensions, respectively. The ^1^H carrier was set to H_2_O resonance, while the ^15^N carrier was centered at 116.85 ppm. NMR resonance assignments were taken from BMRB database accession #4132^28^ and verified by standard triple resonance experiments. Ac-Ubc9_K65_ resonance assignments were obtained by a 3D-HNCA experiment. Average chemical shift perturbation (*Δδ*_*NH*_) was calculated as $\Delta \delta NH=\sqrt{{(5\ast \Delta \delta H)}^{2}+{(\Delta \delta N)}^{2}}$, where *Δδ*_*H*_ and *Δδ*_*N*_ represent the chemical shift change in the amide proton and nitrogen, respectively^8^.

### Modeling the Ubc9-NDSM complex structure

We have previously used HADDOCK 2.1^29^ software to interpret sparse NMR data to build models of Ubc9-NDSM complexes. Docking was performed using the high resolution crystal structure of Ubc9 (PDB_2GRNa) and ambiguous iterative restraints derived from the NMR chemical shift perturbation and knowledge-based interpretation of the sumoylation assay performed on the Ubc9 alanine mutants. We also constructed the model structure of the Ubc9-Calpain2_385-406_ complex using the domain structure of Calpain2_355-514_ known as (PDB_1KFU) to build a more accurate structural model (Supplementary Fig. S1). RanGAP1 is known to bind well to Ubc9, whereas there are no known binding structures for the other NDSM substrates at the sumoylation domain. We performed a protein-protein docking simulation between Ubc9 and the Calpain2 domain using cluspro2.0 software^30^ with restraints between Ubc9 and the SM. Then, we performed molecular dynamic (MD) simulations to optimize the complex structure of the Ubc9-Calpain2 domain (Supplementary Fig. S1) before we employed the Calpain2 peptide (sequence: PQYLIKLEEEDEDEEDGESGCT) from the Calpain2 domain for our purposes.

### *In silico* all-atom molecular dynamic simulations

All-atom molecular dynamic (MD) simulations with explicit water molecules were performed using the crystal structure of Ubc9 (PDB_2GRN) and PMEMD. CUDA^31^ in the AMBER14 MD simulation package with the ff99SB force field^32^. The starting protein system was explicitly solvated with TIP3P water molecules in the rectangular box where the distance to the edge of the solvent box from the protein was chosen to be 16 Å, and the periodic boundary condition was applied. Three sodium ions were added to neutralize the system. The particle mesh Ewald method was applied to treat long-range electrostatic interactions, and a 9.0 Å force-shifted cutoff was used for short-range non-bonded interactions. The hydrogen atoms were constrained to have the equilibrium bond length using the SHAKE algorithm. We performed 2,000 steps of steepest decent minimization followed by 2,000 steps of conjugate gradient minimization. The systems were subsequently subjected to a 10 ps heating process in which the temperature was raised gradually from 30 K to 298 K under the SHAKE algorithm^33^. After the heating step, production runs were carried out for 200 ns with a 2 fs time step and with the NPT ensemble, i.e., constant number of particles (N, ~46,000 atoms), pressure (P, 1 atm), and temperature (T, 298 K). Temperature and pressure were controlled by a Langevin dynamic thermostat with collision frequency of 1 ps^−1^ and a weak-coupling barostat with a coupling constant of 1.0 ps. All trajectories were recorded every 10 ps. The main MD Ubc9 WT and K65 acetylated protein simulations were performed for 10 independent trajectories at up to a 200 ns time scale. Peptide bound Ubc9 was also subjected to the MD simulation under the same conditions as those of Ubc9_WT_ and Ace-Ubc9_K65_. The peptides were RanGAP1_517-536_, Elk1_242-261_, and Calpain2_385-406_, and the peptide bounded structures came from experimental-guided HADDOCK modelling. We also made the mutants of the ×1– ×4 residues to alanine in the Calpain2 peptide from the Calpain2 domain structure and performed MD simulations of five trajectories for each WT Ubc9, K65-acetylated UBC9, and mutants up to 200 ns. The ensembles of the binding structures were sampled by conformational clustering based on the peptide structures (Supplementary Fig. S2). The MD trajectories were analyzed by CPPTRJ in AmberTools15^31^. VMD software^34^ was used to prepare the figures.

## Electronic supplementary material

Supplementary Information
