# Author Correction: Molecular mechanism of K65 acetylation-induced attenuation of Ubc9 and the NDSM interaction

**DOI:** 10.1038/s41598-018-23157-0

**Published:** 2018-03-19

**Authors:** Mandar T. Naik, Mooseok Kang, Chun-Chen Ho, Pei-Hsin Liao, Yung-Lin Hsieh, Nandita M. Naik, Szu-Huan Wang, Iksoo Chang, Hsiu-Ming Shih, Tai-Huang Huang

**Affiliations:** 1 Institute of Biomedical Sciences, Academia Sinica, Taipei, 11529 Taiwan; 2 Department of Molecular Pharmacology, Physiology and Biotechnology, Brown University, Providence, Rhode Island 02903 USA; 3 Department of Physics, Pusan National University, Busan, 46241 Korea; 4 Center for Proteome Biophysics, Department of Brain and Cognitive Sciences, DGIST, Daegu, 42988 Korea; 5 Institute of Molecular and Genomic Medicine, National Health Research Institutes, Miaoli County, 35053 Taiwan

Correction to: *Scientific Reports* 10.1038/s41598-017-17465-0, published online 12 December 2017

This Article contains an error in the Author Contribution section.

“M.T.N. acquired and analyzed N.M.R. data, performed structure modelling and data analysis, and wrote the paper; M.K. performed M.D. simulation and data analysis; C.-C.H., P.-H.L., Y.-L.H. and N.M.N. did wet lab work; S.-H.W. acquired and analyzed ITC data. I.C. designed M.D. 25 simulation project, performed data analysis, and wrote the paper. H.M.S. and T.H.H. conceived and designed the experiments, and wrote the paper.”

should read:

“MTN acquired and analyzed NMR data, performed structure modelling and data analysis, and wrote the paper; MK performed MD simulation and data analysis; C-CH, P-HL, Y-LH, S-HW, and NMN did wet lab work. IC designed MD 25 simulation project, performed data analysis, and wrote the paper. HMS and THH conceived and designed the experiments, and wrote the paper”

