# Supplementary material for: Molecular mechanism of K65 acetylation-induced attenuation of Ubc9 and the NDSM interaction
Source: Sci Rep. 2017 Dec 12;7:17391. doi: 10.1038/s41598-017-17465-0 (PMC5727262; doi:10.1038/s41598-017-17465-0)
Supplement: Supplementary file 1 — Supplementary Information [file 41598_2017_17465_MOESM1_ESM.pdf]

Molecular mechanism of K65 acetylation-induced attenuation of Ubc9 and the NDSM interaction

Mandar T. Naik[1, 2, #], Mooseok Kang[3, #], Chun-Chen Ho[1], Pei-Hsin Liao[1], Yung-Lin Hsie[1], Nandita M. Naik[1], Szu-Huan Wang[1], Iksoo Chang[4, *], Hsiu-Ming Shih[1, *], Tai-Huang Huang[1, *]

Supplementary Table 1

Supplementary table T1. The hydrogen-bond occupancy between the wild and the K65-acetylated UBC9+Calpain2, UBC9+ELK1, UBC9+CBP. It shows the importance of the second negative residue.

NDSM	Acetylation	@Ubc9	x1	x2	x3	x4	x5	x6	x7	x8	x9	x10
Calpain2	Residue types		E	E	D	E	D	E	E	D	G	E
	WT	K65	1.47	8.44	0.00	0.00	0.00	0.00	0.00	0.00	0.00	0.11
		K74	0.02	78.33	0.00	0.04	0.00	0.00	0.00	0.00	0.00	0.00
		K76	0.00	0.04	0.00	69.91	0.00	0.00	0.00	0.00	0.00	0.00
	K65ac	acK65	0.00	0.02	0.00	0.00	0.00	0.03	0.00	0.00	0.00	0.04
		K74	0.00	1.77	0.00	0.00	0.00	0.00	0.00	0.00	0.00	0.48
		K76	0.00	2.73	0.00	0.00	0.00	0.00	0.00	0.00	0.00	0.00
Elk1	Residue types		G	P	K	E	E	L	E	V	A	G
	WT	K65	0.00	0.00	0.00	28.70	7.22	0.00	0.39	0.00	0.00	0.03
		K74	0.00	0.00	0.00	1.74	64.88	0.00	2.09	0.00	0.00	0.00
		K76	0.00	0.00	0.00	1.56	44.27	0.00	8.55	0.00	0.00	0.00
	K65ac	acK65	0.00	0.00	0.00	0.00	0.00	0.00	0.00	0.00	0.00	0.00
		K74	0.00	0.00	0.00	0.00	0.00	0.00	0.00	0.00	0.00	0.00
		K76	0.00	0.00	0.00	0.33	0.00	0.00	76.31	0.00	0.00	0.00
CBP	Residue types		V	K	E	E	E	E	S	S	S	N
	WT	K65	0.00	0.00	0.00	0.18	0.39	1.03	0.00	0.18	1.09	4.37
		K74	0.00	0.00	0.00	76.94	0.06	0.48	22.33	8.77	0.00	0.00
		K76	0.00	0.00	0.10	48.79	0.02	0.38	0.00	0.25	0.00	0.00
	K65ac	acK65	0.00	0.98	0.00	0.01	0.00	0.00	0.00	0.00	0.00	0.00
		K74	0.00	0.00	73.26	0.03	0.56	0.00	0.00	0.09	0.00	0.00
		K76	0.00	0.00	8.35	0.00	91.54	0.00	59.22	37.75	0.00	0.00

(Hydrogen-bond occupancy, %)

Molecular mechanism of K65 acetylation-induced attenuation of Ubc9 and the NDSM interaction

Mandar T. Naik[1, 2, #], Mooseok Kang[3, #], Chun-Chen Ho[1], Pei-Hsin Liao[1], Yung-Lin Hsie[1], Nandita M. Naik[1], Szu-Huan Wang[1], Iksoo Chang[4, *], Hsiu-Ming Shih[1, *], Tai-Huang Huang[1, *]

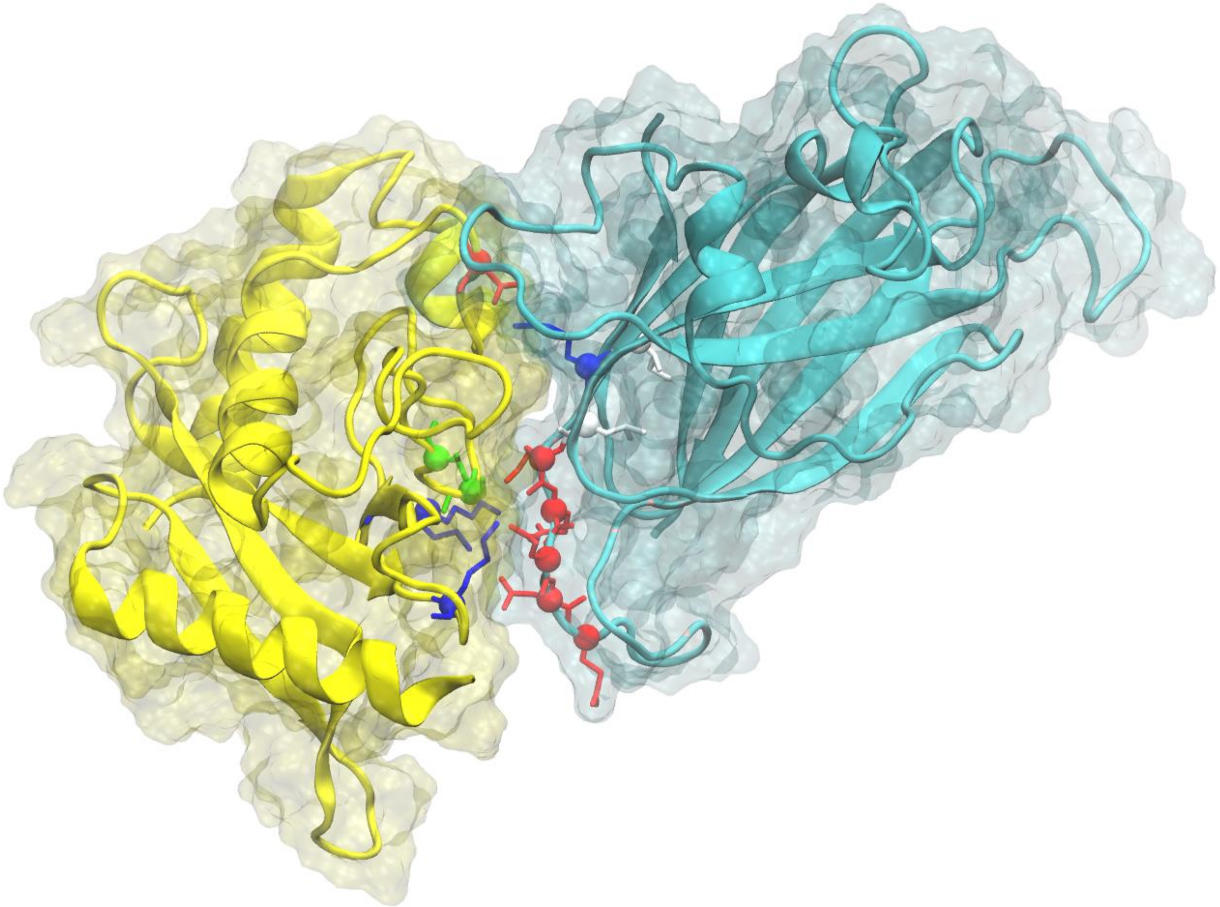

Supplemental figure S1. Modeled structure of Ubc9 (yellow)-Calpain2$_{355\text{-}514}$ Domain (cyan) complex. The sumoylation residues are shown by a stick and a ball, and the color represents the residue property. (Red : negatively charged, Blue : positively charged , Green : polar uncharged, White : hydrophobic)

Molecular mechanism of K65 acetylation-induced attenuation of Ubc9 and the NDSM interaction

Mandar T. Naik[1, 2, #], Mooseok Kang[3, #], Chun-Chen Ho[1], Pei-Hsin Liao[1], Yung-Lin Hsie[1], Nandita M. Naik[1], Szu-Huan Wang[1], Iksoo Chang[4, *], Hsiu-Ming Shih[1, *], Tai-Huang Huang[1, *]

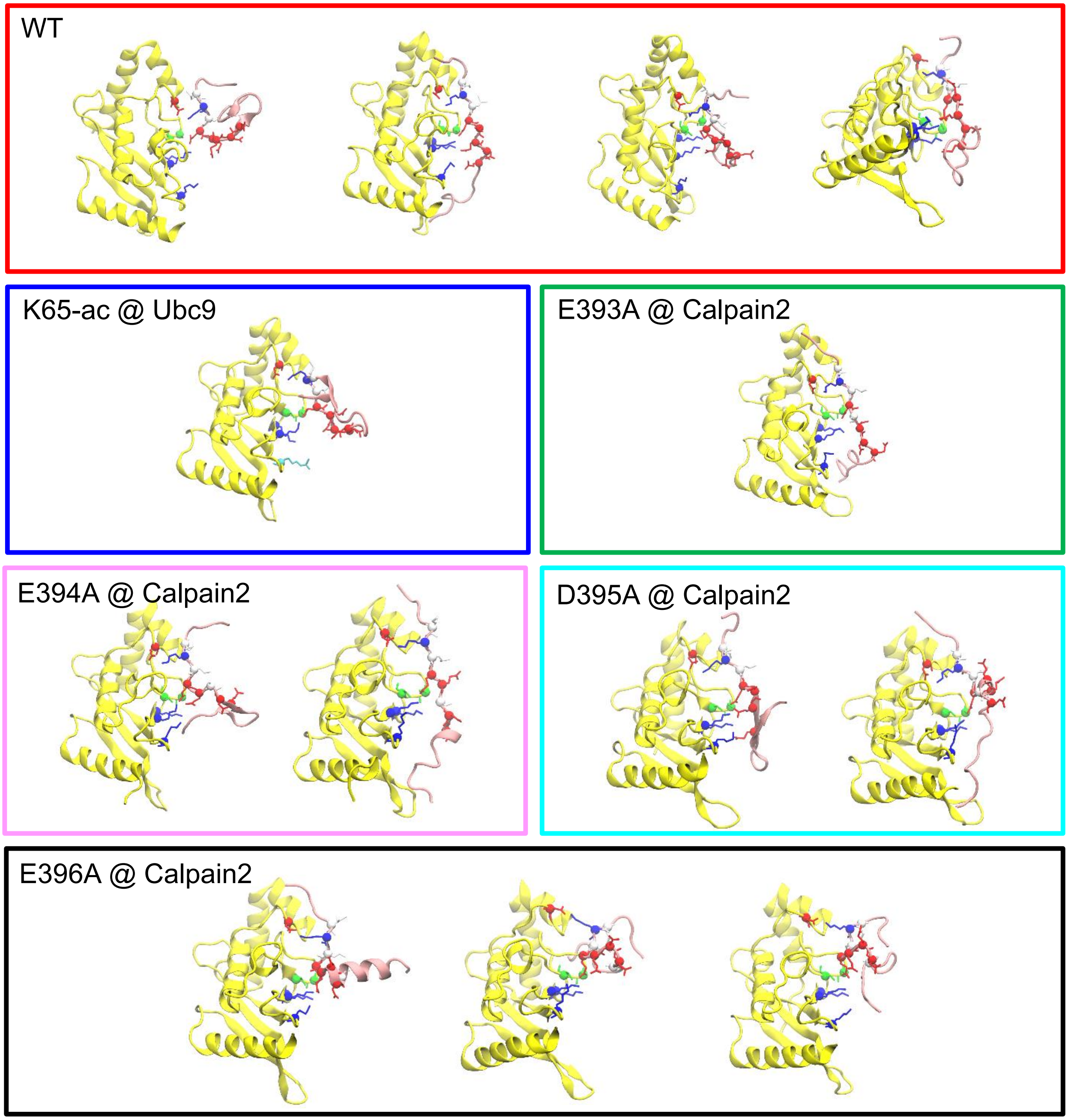

Supplemental figure S2. Clustering of binding structures of Ubc9-Calpain2 peptide complex
